# Increasing rat numbers in cities are linked to climate warming, urbanization, and human population

**DOI:** 10.1126/sciadv.ads6782

**Published:** 2025-01-31

**Authors:** Jonathan L. Richardson, Elizabeth P. McCoy, Nicholas Parlavecchio, Ryan Szykowny, Eli Beech-Brown, Jan A. Buijs, Jacqueline Buckley, Robert M. Corrigan, Federico Costa, Ray DeLaney, Rachel Denny, Leah Helms, Wade Lee, Maureen H. Murray, Claudia Riegel, Fabio N. Souza, John Ulrich, Adena Why, Yasushi Kiyokawa

**Affiliations:** ^1^ Department of Biology, University of Richmond, 138 UR Drive, Richmond, VA 23173, USA.; ^2^ Municipal Health Service, Amsterdam, Netherlands.; ^3^ Urban Wildlife Institute, Lincoln Park Zoo, Chicago, IL, USA.; ^4^ RMC Pest Management Consulting, Chappaqua, NY, USA.; ^5^ Instituto de Saúde Coletiva, Universidade Federal da Bahia, Salvador, Brazil.; ^6^ City of Philadelphia, Department of Public Health, Environmental Health Services, PA, USA.; ^7^ New Orleans Mosquito, Termite, and Rodent Control Board, New Orleans, LA, USA.; ^8^ Seattle/King County Solid Waste, Rodent, and Zoonotic Disease Program, Seattle, WA, USA.; ^9^ Alameda County Department of Environmental Health, Alameda, CA, USA.; ^10^ City of Boston, Department of Inspectional Services, Boston, MA, USA.; ^11^ Laboratory of Veterinary Ethology, The University of Tokyo, Tokyo, Japan.

## Abstract

Urban rats are commensal pests that thrive in cities by exploiting the resources accompanying large human populations. Identifying long-term trends in rat numbers and how they are shaped by environmental changes is critical for understanding their ecology, and projecting future vulnerabilities and mitigation needs. Here, we use public complaint and inspection data from 16 cities around the world to estimate trends in rat populations. Eleven of 16 cities (69%) had significant increasing trends in rat numbers, including Washington D.C., New York, and Amsterdam. Just three cities experienced declines. Cities experiencing greater temperature increases over time saw larger increases in rats. Cities with more dense human populations and more urbanization also saw larger increases in rats. Warming temperatures and more people living in cities may be expanding the seasonal activity periods and food availability for urban rats. Cities will have to integrate the biological impacts of these variables into future management strategies.

## Teaser

Most cities are seeing increases in rat numbers, linked to climate warming, urbanization, and human population densities.

## INTRODUCTION

Commensal rats in the genus *Rattus* are among the most ubiquitous and important pest species. Two species (*Rattus norvegicus* and *Rattus rattus*) have near-global distributions, now occurring in every continent except Antarctica. Rats damage infrastructure, consume agricultural yields, and contaminate food supplies, causing an estimated US$27 billion in damage each year in the United States alone (*1*). Rats also harbor and transmit more than 50 zoonotic pathogens and parasites to people, affecting public health around the world (*2*, *3*). Associated diseases include leptospirosis, hantavirus pulmonary syndrome, murine typhus, and bubonic plague. Rats thrive in human-dominated landscapes by exploiting resources concentrated where human population density is high (*4*) and are often classified as urban exploiting species. As a result, rat population densities are expected to be higher in cities than in rural areas, with the potential to negatively affect more people (*5*). The very presence of rats also takes a measurable toll on the mental health of people living in contact with them (*6*).

Municipalities and property owners have been trying to reduce rat numbers for centuries. In recent decades, efforts at suppressing or eradicating rats have primarily been through the use of lethal rodenticide chemicals or traps rather than nonlethal options that would make the environment less suitable (e.g., securing food waste and removing harborage) (*7*). Globally, the control efforts associated with this “war on rats” cost an estimated US$500 million every year (*8*). At the municipal level, the strategies and intensity of these control efforts vary widely among cities. Rodent control is also inconsistent within cities over time, as priorities shift, budgets and staff fluctuate, and new control products or approaches are introduced.

One of the most intractable challenges associated with rodent control is tracking rat numbers in a consistent way over time, which is a necessary step to assess whether control efforts are effective (*9*). The common presumption cited in many media reports is that rat numbers are increasing around the world. Yet, rarely are formal scientific population surveys done for urban rats, as city budgets and agencies struggle to simply keep up with responding to rat complaints and infestations. Because of the absence of consistent long-term data, we are no closer to understanding the effectiveness of rat population control efforts (*7*). Tracking rat numbers over time is also needed for any basic understanding of the demography and population ecology of these urban rat populations, for which there has been little research on over the past 70 years (*10*).

While untested, the assertion that rat populations are growing is in line with potential biological responses to changing urban environments. As small mammals, rats must maintain internal body homeostasis and are limited by cold temperatures during winter (*11*, *12*). Warming temperatures resulting from climate change or urban heat islands may extend the seasonal window for aboveground foraging and active breeding period for rats, supporting population growth (*13*). Increasing human population size and urbanization are also likely to provide more food waste as a resource and structural habitats that support rat populations. As rapid urbanization continues, it is critically important to track rat numbers and assess both the changes in their population ecology and our progress in controlling them. The human population living in cities is projected to increase 25% by 2050 (from 56% in 2020; World Bank), and total urban land cover across the world is also projected to increase 185% between the years 2000 and 2030 (*14*), providing even more suitable habitat and food waste for urban rats. This suggests that cities will become increasingly conducive environments for rats, but data are needed to confirm these effects and quantify their impact on rat numbers.

In this study, we use between 7 and 17 years (average of 12.2 years) of public rat sighting and inspection data from 16 cities around the world to quantify changes in rat numbers for each city and to evaluate trends across cities. Rat sightings coming from the public correlate well with relative abundance measures from trapping (*15*–*17*) and are an important proxy for rat numbers. We accessed rat reporting data for large US cities where such data are collected and available, or that we could request from cities that do not publicly report such data. To expand the geographic scope, we also requested data from other cities and rat researchers outside of the US, where public data collection is limited or not made available to the public. We limited our analyses to cities where the data collection methods and systems remained largely consistent during the study period to avoid possible trends stemming from changing data intake methods. We also assessed whether several relevant variables were linked with trends in rat numbers: human population density, ambient temperature changes over time, annual minimum temperatures, levels of urbanization, and socioeconomic variation among cities (see full Materials and Methods below). For reasons related to the biology of *Rattus* species, we hypothesized that most cities are experiencing an increasing trend in rat numbers and that rats are increasing fastest in larger cities with (i) more dense human populations, (ii) warmer winter temperatures, (iii) steeper temperature increases over time, (iv) less vegetation and greater urbanization, and (v) lower gross domestic product (GDP), as a proxy of socioeconomic resources available to implement rat control efforts.

## RESULTS

**Fig. 1.**
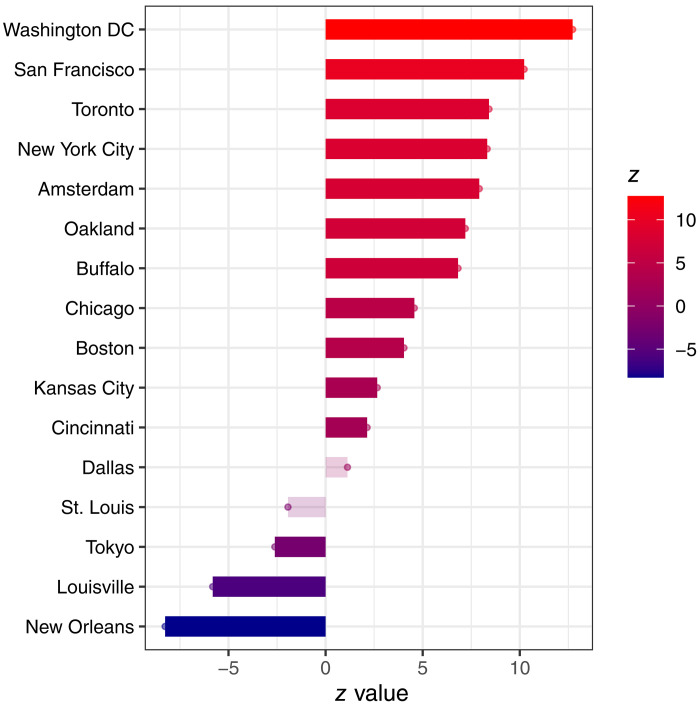
Trends in rat sightings across 16 cities. Mann-Kendall trend test statistic, estimating changes in rat numbers for 16 cities that have long-term data on public rat complaints and municipal inspections. Positive *z* values represent increasing rat numbers over time, and negative values are decreasing rat trends. All cities had a significant trend (increase or decrease) except for Dallas, Texas, and St. Louis, Missouri, USA, denoted with transparent bars. Note that the *z* value does not represent the raw numbers of rats observed but rather the change in these numbers over time.

For 11 of the 16 cities (69%) in our dataset, rat numbers significantly increased during the study period (Fig. 1). Washington, D.C., San Francisco, Toronto, New York City, and Amsterdam exhibited the five strongest positive trends, followed by Oakland, Buffalo, Chicago, Boston, Kansas City, and Cincinnati. The magnitude of trends among cities varied widely; for example, the trend in rat numbers in Washington, D.C. was three times greater than in Boston and 1.5 times greater than New York City. In contrast, Tokyo, Louisville, and New Orleans each had declining trends in rat numbers, with New Orleans experiencing the greatest decrease over the study period (Fig. 1). Dallas and Saint Louis did not show significant trends over time.

**Fig. 2.**
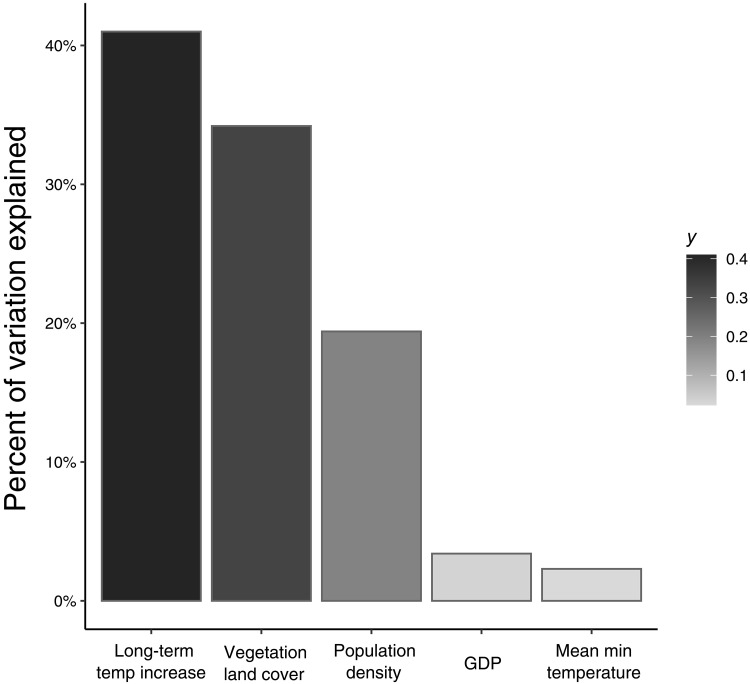
Relative contribution of environmental and social variables on rat trends. Scaling correlation coefficients within one model, a relative weights analysis found that the strength of each city’s rat trends were most strongly linked to the mean temperature increase experienced in each city over the past century (40.7% of variation). The proportion of vegetation cover within each city (a proxy of urbanization) explained 34.3% of the variation in rat numbers over time, while human population density accounted for 19.4%. GDP (3.4%) and mean annual minimum temperature (2.3% of variation) were each less associated with rat trends.

**Fig. 3.**
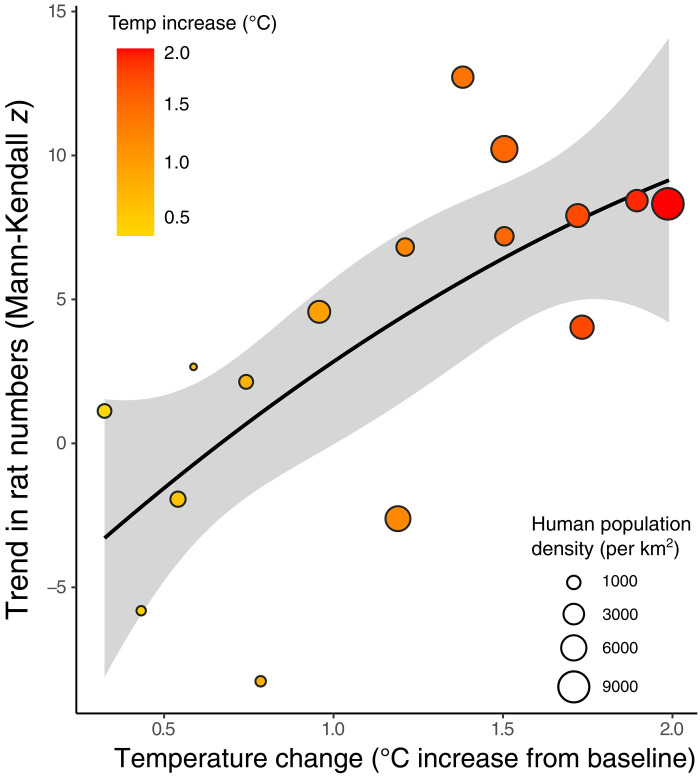
Positive association between warming temperatures and rat numbers. Increasing trends in rat numbers are associated with the mean temperature increase experienced in each city, above long-term baseline mean temperatures (*r*^2^ = 0.478, *P* = 0.003). Each data point represents one city, and the size of each point corresponds to the human population density within that city, which was also associated with the trend in rat numbers (*r*^2^ = 0.29, *P* = 0.031).

In a relative weights analysis, 40.7% of the variation in trend strength was linked to the mean temperature increase a city had experienced relative to long-term temperature averages (Fig. 2). Cities that had a greater rise in temperature over time had larger increases in rat sightings (Fig. 3). The percentage of a city’s land area that was vegetated (a proxy of urbanization) had a relative weight value of 34.3%, where cities with less vegetation experienced greater increases in rats. Human population density had a weight of 19.4%, followed by GDP (3.4%), and mean minimum temperature experienced by a city (2.3%). Overall, 66% of all variation in the trend data was explained by these five explanatory variables.

**Fig. 4.**
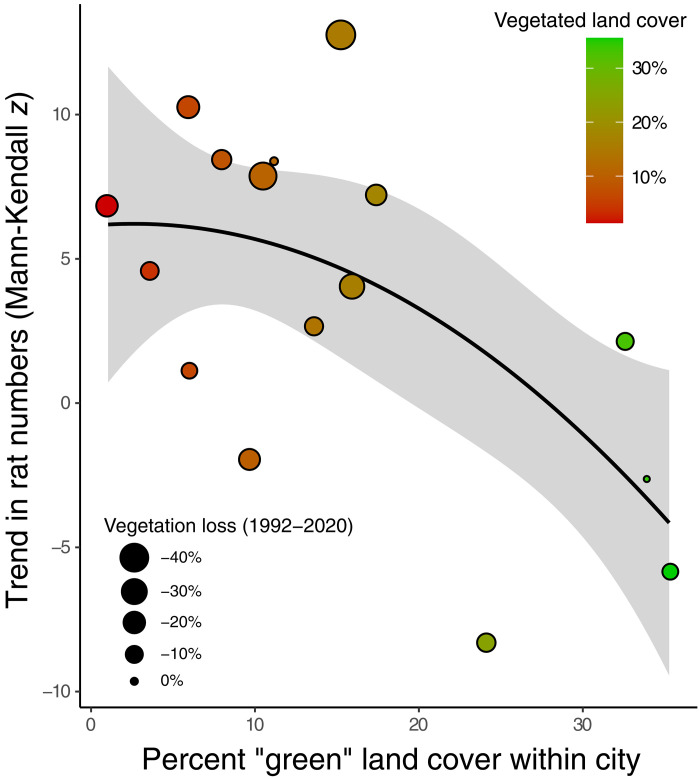
Negative association between vegetation cover and rat numbers. Increasing trends in rat numbers are associated with the percentage of a city’s land area that is vegetated (2020 data: *r*^2^ = 0.346, *P* = 0.017), which is a proxy for the degree of urbanization. Each data point represents one city, and the size of each point corresponds to the amount of green land cover that was lost between the years 1992 and 2020, which is a measure of the rate of urbanization, and was also associated with the trend in rat numbers (*r*^2^ = 0.268, *P* = 0.04).

Separate regression analysis also found that the long-term temperature trend was strongly associated with rat numbers, with cities that have experienced the greatest warming (over long-term baseline temperatures) also experiencing faster increases in rat numbers (*r*^2^ = 0.478, *P* = 0.003; Fig. 3). Cities that have a higher percentage of green land cover experienced the inverse pattern, with slower growing or even decreasing rat numbers (*r*^2^ = 0.346, *P* = 0.017; Fig. 4). The human population density was positively correlated to rat trends (*r*^2^ = 0.29; *P* = 0.031). In addition, the change in vegetation cover between the years 1992 and 2020, which is a proxy for urbanization rates, was also associated with rat trends (*r*^2^ = 0.268; *P* = 0.04), meaning that cities that lost more vegetated areas (and became more urbanized) during the period saw greater increases in rat numbers. The trends in rat numbers were not linked to GDP (*r*^2^ = 0.057; *P* = 0.369) or mean minimum temperature in each city (*r*^2^ = 0. 027; *P* = 0.543).

## DISCUSSION

### Rat trends linked to climate warming

The environmental variable that was most strongly linked to increases in rats was the change in temperature experienced by each city relative to long-term baseline average temperatures. Cities that had greater increases in average air temperatures had larger increases in their rat numbers (Fig. 3). As with most small mammals, rat activity is constrained by colder temperatures. When temperatures decrease, the thermal physiology of rodents means that they either have to remain sheltered longer or forage more food to maintain thermal homeostasis via higher metabolism (*18*). At northern latitudes, *R. norvegicus* show strong seasonality in abundance, activity, and reproductive output (*19*–*23*), whereas minimal seasonality is observed in tropical and subtropical climates (*24*–*26*). For example, rats in New York City exhibit consistent seasonal cycling across years, with peak numbers in late summer and a nadir during the middle of winter (*23*). Other small rodent species show related latitudinal variation in abundance and success, as Bai *et al.* (*27*) found that the native Brant’s voles in the southern part of their range in China were less successful with warming temperatures than northern populations, leading to range contraction due to the physiological constraints of the critical thermal limits of this species (*27*). In laboratory colonies of *R. norvegicus*, cold temperature can induce stress responses, with enlarged adrenal glands, reduced food consumption, and signs of anxiety (*28*). Furthermore, *R. rattus* is primarily an aboveground species, and their global distribution is likely limited by a combination of harsh winter temperatures and the presence of arboreal competitors [e.g., squirrels; (*29*, *30*)].

Warmer temperatures, particularly during cooler seasons of the year, may release rats from physio-thermal limitations. This can be due to a combination of lower winter mortality, longer periods of aboveground activity and foraging, and increased fecundity. While wild male rats produce sperm throughout the year (*31*), Perry (*32*) found pregnancy rates to be lowest in cold months in Liverpool, England, and Davis *et al.* (*10*) found a similar pattern in Baltimore, USA. In New York City, we have seen higher numbers of rats during February and March outdoor inspections over the past 5 years relative to previous years. However, there was no association between rat increases and the mean minimum temperature for the coldest periods in the 16 cities of this study (*r*^2^ = 0. 027; *P* = 0.543). At a larger scale, there is evidence that *R. rattus* may be expanding its geographic range in response to current warming climates. Harris *et al.* (*33*) document an elevational expansion of black rats in New Zealand since 2007, linked with warming winters. Bai *et al.* (*34*) found that the range expansion of *Rattus tanezumi* in China over the past century was linked with increasing temperatures and human density. These trends not only have implications for ecological range dynamics and invasive pest management but potentially for increased zoonotic disease risk, as well (*35*). For example, rat-borne leptospirosis infections may increase as rats are active longer, and the *Leptospira* bacteria are more likely to persist in standing water than in what previously would have been ice.

Long-term climate warming is occurring across much of the planet. Warming is projected to be more intense in cities (*36*), where the urban heat island effect already produces higher temperatures than in surrounding rural areas. Climate models project urban temperatures to rise 1.9° to 4.4°C by the year 2100 based on forecasts of greenhouse gas emissions. Furthermore, this warming is not happening uniformly across the globe. Urban areas of northern North America, southern and central Europe, and the Middle East are projected to have faster increases in temperature (*37*), and this may lead to cities in these regions experiencing different trends in rat numbers over time, as well as the associated human-rat conflicts.

Our study found that cities where temperatures are increasing faster had larger increases in rat activity and sightings (Fig. 3). It is not clear from our data whether this increase is because rats have lower mortality, increased fecundity, or increased foraging opportunities, although each of those mechanisms is related to the potential for rats to increase their activity during cooler months due to warming. However, in the 16 cities assessed in our study, there was no correlation between monthly mean temperature and the *z* value trend for each month. In other words, a regression analysis done with each city’s monthly mean temperature across years and the *z* value for separate months found no association, (i.e., January trend, February trend, etc. were not different from each other; *P* = 0.169), meaning that the increase in sightings in a winter month was not greater than a summer month. There was also not a strong relationship between a city’s lowest mean wintertime temperature and the rat trend *z* statistic, suggesting that cities with colder winters are not experiencing a larger increase in rats over time than warmer cities even if activity and population structure changes during the winter (*23*, *38*). However, there are notably no cities among our 16 in tropical climate zones, and only 1 subtropical location (New Orleans). Insights from nontemperate cities nearer to the equator will be important to fully understand the latitudinal climate links to rat population dynamics. Given the projections of continued warming for the foreseeable future, cities need to be prepared for the potential for this warming to exacerbate current rodent pest infestation levels. More financial and personnel resources will need to be dedicated to municipal rodent control efforts to limit this expected increase in rat populations and activity.

### Urbanization and greenspace availability associated with rat trends

We found that cities that had less vegetated land cover and, hence, more urbanization, experienced increased growth of their rat numbers (Fig. 4). This may be related to both the habitat preferences of rats and food availability, and how those both vary across a city. There is debate in the field of urban ecology about how vegetation, greenspaces, and formal parks influence rat abundance. While rats (particularly *R. norvegicus*) benefit from access to bare ground and soil for their burrowing habits, large greenspaces also have less food waste resource availability. *R. norvegicus* is also able to use microhabitats of bare soil (e.g., street tree pit) or nest within cluttered harborage (e.g., discarded furniture or shipping pallets) that is likely to be closer to a consistent food resource (e.g., restaurant garbage bin). Previous studies have found rats both positively and negatively associated with vegetated greenspaces. For example, rat complaints in Tokyo were negatively correlated with proximity to greenspaces (*39*), while two studies found that evidence of rats in New York City went down in areas farther from public open spaces and vacant lots (*40*, *41*). A study looking at three cities in the Netherlands found a positive association between “greenness” (i.e., normalized difference vegetation index) and rat abundance (*17*).

At a smaller spatial scale, the presence of vegetation also provides cover for rats to move around with less fear of being detected by predators, and has been associated with rat abundance. Rats were more likely to be found in vegetated areas of Salzburg, Austria (*42*, *43*), and the presence of ground plantings and dense shrubbery was associated with more Norway rat activity in Boston, USA (*44*). However, in our study, we were comparing broad-scale differences between the 16 cities, which required global data on land cover. The coarse resolution of the data means that we were looking at broad patterns of urbanization in these cities and are unable to resolve how microhabitat differences in vegetation may influence rats over time compared to broader patterns of greenness and potential shading or thermal moderation.

The rates of vegetation loss and urbanization were also associated with trends in rat numbers, with the caveat that there was collinearity between this temporal change variable and the 2020 snapshot value of land cover. Cities that lost more vegetation between the years 1992 and 2020 experienced larger increases in rats. This may be due to an increase in the availability of new urban infrastructure that is more suitable habitat for rats. This includes more residences, food service establishments, and refuse generated as a result. A loss of vegetation in cities will also likely reduce shading and increase heat retention, exacerbating the urban heat island effect. However, there was no correlation between the loss of vegetation (1992 to 2020) and the degree of warming across the 16 cities (*r*^2^ = 0.10; *P* = 0.231). The data in this study suggest that preserving vegetated greenspaces and restricting urbanization may help cities slow the increase in rats, in combination with other strategies (see below).

### Human population correlated with increases in rats

Across the 16 cities assessed, greater human population density was associated with larger growth in rat numbers (*r*^2^ = 0.29; *P* = 0.031). As more people inhabit a city, more food waste becomes available as a resource for rats. In addition, more urban infrastructure (e.g., buildings, small parks, and underground utilities) will also be available for rats to use and occupy as harborage and habitat. At smaller scales within cities, rat presence and abundance has been linked to human density. Sánchez *et al.* (*45*) found a positive association between density of people and public rat complaints in Chicago, USA, yet there was no similar association in New York City (*40*). Evidence of active rat signs in New York City did, however, increase in areas with higher densities of residential units and restaurants (*41*), and a similar association was found with rat abundance and human density and food serving establishments in Barcelona (*16*). In Amsterdam, a previous study found that rat abundance was linked with the number of inhabitants within a neighborhood, as was the percentage of buildings constructed before 1960 and the percentage of greenspace (*46*). Among the studies, we can glean insights on rat habitat preferences within cities.

There was no association between per capita GDP economic output for each city and trends in rat numbers (*r*^2^ = 0.057; *P* = 0.369). GDP can translate to a larger tax base, which cities could allocate to rodent management programs, or higher socioeconomic resources that could be used by private property owners to hire and implement pest control services. However, the lack of a relationship suggests that more affluent, well-resourced cities are seeing the same levels of rat population increases as poorer cities. More research is needed to determine whether this is due to the resources being allocated to pest control, the operations decisions regarding how to implement controls, or some other suite of variables that differs across cities.

### Impacts of climate change and urbanization on rat populations in cities

The human population is projected to grow by more than 20% by 2050 (*47*) (United Nations), and most of that growth will occur in cities (World Bank). The growing urban population will also lead to nearly a doubling of urban land cover across the globe in the first half of the 21st century (*14*), equal to 1,200,000 km^2^ of newly converted urban land cover by 2030 (*48*) (World Bank). This expanding urban footprint, and the built infrastructure associated with it, creates new habitat and generates more food resources for commensal urban exploiting species like *Rattus* species. The data from our study suggest that growing urban populations do play a key role in increasing rat numbers but that warming temperatures associated with climate change may be an even stronger driver of rat population growth in cities around the world.

The average rate of warming across the globe has been 0.2°C per decade since 1975, and that rate of climate warming is accelerating (Intergovernmental Panel on Climate Change). However, the rate of warming is not uniform across the world. Cities already experience warmer temperatures than their surrounding areas through the urban heat island effect (*49*). Our study of 16 cities indicates that continued warming temperatures may drive further increases in urban rat populations, likely through releasing them from thermal physiological limitations and supporting longer reproductive windows and higher fecundity. This may occur until an upper thermal limit is eventually reached at excessively high temperatures, where rats may experience reduced fitness via fetal anomalies (*50*), cardiac damage (*51*), and lower fertility (*52*, *53*).

### Potential ecological impacts of increasing rats

Increases in rat numbers may alter urban food webs, which are generally less complex with fewer trophic levels than nonurban ecosystems (*54*), in several ways. While there is no evidence that rats are a primary food resource for urban predators, they are known prey for several mesopredators like coyotes and birds of prey. Therefore, increasing rat numbers may supplement the diet of these predator species and lead to higher survival and reproduction rates and possible larger population sizes of those species. Yet, Magle *et al.* (*55*) found that predator species still avoid high-density urban areas, creating a spatial mismatch between rats as prey and their potential predators. Related to domestic predators, Parsons *et al.* (*56*) documented less than a 1% predation rate between feral cat carnivores and rats in an urban setting. However, when animals do consume urban rats that have consumed toxic rodenticides, these toxicants often accumulate in and harm the nontarget predators (*57*). Commensal rats also primarily feed on human food waste, which in cities can serve as a detritus base of urban food webs (*58*). It has been suggested that commensal pests like rodents act as a relevant detritivore on this waste, removing some portion of it from urban systems. In addition, while most of the commensal rat diet is plant material (*59*), some urban rats can have higher proportions of arthropods and worms in their diets (*60*). Further work would be needed to know just how an increasing rat population could affect other trophic levels within the urban food web.

### Implications for controlling rat numbers in cities

Controlling climate change itself requires international collective regulations to limit increased warming, which is outside of the ability of individual cities. Furthermore, slowing human population growth in cities is also unlikely, given the global trends of people shifting to a more urban distribution. Therefore, the management of urban rats will need to focus on aggressive strategies that cities can implement to slow the increase of rat numbers that is likely to continue. These strategies include (i) modern refuse and food waste management practices (e.g., rodent-proof trash containers/dumpsters, frequent garbage collections, and food waste diversion programs), (ii) enacting and enforcing regulations related to rodent exclusion building codes and timely removal of loose materials used for harborage (i.e., clutter), and (iii) devoting more resources to lethal and nonlethal control, public education, and surveillance for areas of infestation across the city that will require intensive intervention efforts (*61*). Lessons can also be gleaned from the few cities in this study that had negative trends in rat numbers. For example, the New Orleans department tasked with rodent control conducts proactive surveillance of rat activity and has increased efforts to engage other city departments and residents in education and control options. Tokyo has high cultural expectations of hygiene that promote sanitation standards, which has been amplified by social networking/media platforms where people can quickly publicize unsanitary conditions and rats they see in the city.

The most promising integrated pest management (IPM) strategies focus on making the urban environment less conducive for rats rather than outright removal of rats already there. Cities and the relevant stakeholders will need to fully support these different components of an IPM approach, moving away from the decades-long dependence on rodent poisons, which have had limited long-term success and important negative impacts on the environment, nontarget wildlife, and even the genetics of the target rat population (*7*, *62*, *63*). For example, despite a marked increase in rodenticide application by New York City between 2014 and 2019 (*64*), our analysis found a consistent increase in rat sightings in that city during that same period. An intensive rodenticide application campaign in Salvador, Brazil also yielded minimal reductions in rat numbers (*65*). In a promising early change, in late 2023 and early 2024, the government of New York City implemented new policies requiring buildings in certain neighborhoods to containerize garbage (i.e., no longer leave it in bags on the sidewalks) and moved the earliest time that garbage could be set outside to 4 hours later. Early data from 2024 indicate that the number of rat complaints in those areas of the city may be lower than in recent years.

While the results of our study provide a broad picture of patterns among cities around the world (and at the city-wide level), the control strategies to mitigate these increases in rat numbers will ultimately be implemented across individual cities. That means that it is imperative for cities to develop rodent IPM programs that account for the unique environmental and social heterogeneity that exists across their city and that shape rat abundance within their service areas (*45*). Because of the association between rats and socioeconomic disparities, it is imperative that management policies are implemented equitably across cities. Municipalities that create aggressive control campaigns that skew efforts toward well-resourced, higher socioeconomic areas of a city risk creating a patchwork of “source” and “sink” habitats. In this scenario, unabated rat populations in the most impoverished neighborhoods receiving less management attention can serve as a source of individuals that disperse into wealthier areas of the city receiving more rodent intervention efforts from the city, thereby perpetuating infestation issues. For these reasons, each municipality will need to develop an IPM plan that accommodates the specific environmental and socio-ecological context of that city.

Ultimately, it is unlikely that most cities will ever fully eradicate rats. However, quickly and efficiently implementing systemic IPM strategies can at least abate the increasing numbers documented here and bring rat population sizes to levels that are deemed tolerable and within the cultural carrying capacity (*66*) of each city. Our study also demonstrates the need for cities to rigorously track rat numbers over time to evaluate the effectiveness of their control programs or to identify new infestations in need of acute mitigation. We used public complaints and request for services data for most of the 16 cities in our study because they were the only systematic data available. While rodent complaints correlate closely with rat abundance (*15*–*17*), there are limits and biases to these data, including awareness of reporting options and comfort interacting with government resources. These issues do not affect analyses across entire cities over time (e.g., the current study) but do suggest that our estimates here may even be conservative, making the management implications even more pressing. The limits of public reporting data point to a strong need for municipalities to do rigorous, long-term monitoring initiatives that will provide an independent and less biased estimate of rat numbers and activity. The need for this systematic data, as well as the implementation of aggressive IPM programs, means that cities will have to increase funding and staffing in the relevant agencies and departments if they hope to mitigate rat increases in the coming decades.

## MATERIALS AND METHODS

We acquired rat sightings data primarily via public municipal databases, conducting searches for pest inspection reports and public complaint data. We started this study focusing on cities in the United States, which generally have public complaint reporting systems (e.g., “311” platforms). We looked for publicly available databases in the 200 largest cities (by population size) in the US. Cities typically file each complaint or inspection report into general categories. We sorted each year’s complaints by category, selecting complaints filed under terms such as “Rat Sighting” and “Rat Treatment.” The relevant search terms used for each city’s datasets are listed in table S1. The sorting and filtering process also involved eliminating cities that had noted shifts in reporting methods or data inconsistencies, such as long gaps in data availability. We also eliminated cities that used a generic “pest” category and did not distinguish between rodents and insects, for example. The last data collection phase involved reaching out to cities where the data were unclear or unavailable to request complete information. We excluded any cities that did not have the equivalent of at least 7 years of records. It is less common for cities outside of the US to make their public complaint data available through a public portal. To expand the insights of our study beyond the US, we reached out to researchers and municipal officials in numerous cities around the world. We were able to acquire data that met our criteria for three cities outside of the US.

Public complaint data are not the same as data coming from a systematic survey for rats. However, traditional surveying techniques like trapping or surveys of “active rat signs” (*41*) are rarely conducted by cities, requiring the use of other data sources to assess rat populations to overcome these data limitations (*67*). There are risks of bias in public reporting data if all residents are not equally likely to report a rat sighting based on differences in familiarity with rats, renter status, or socioeconomic or comfort levels interacting with the city government. Yet, studies in Chicago, Barcelona, Amsterdam, Rotterdam, and Eindhoven found that there was a close association between public rat complaints and systematic trapping estimates of relative abundance (*15*–*17*). In addition, in the current study, we were taking total public complaints across the entire city, and across multiple years, to look at the change in numbers, not the absolute numbers, that bypasses any potential risk of within-city heterogeneity or temporal heterogeneity in reporting bias.

After collecting and filtering the necessary data, we tabulated the number of monthly rat reports in each city for as many months as were available. We also acquired data on the human population density and population change via census reporting. Temperature change over historic baseline mean temperatures and mean temperatures were obtained from national weather agency data (e.g., the National Oceanic and Atmospheric Administration in the US and the Royal Netherlands Meteorological Institute in the Netherlands; table S1) and published reports. City-level GDP was used as a measure of municipal socioeconomics, obtained from publicly available reports. More information on these measurements can be found in table S1. We excluded several variables that were correlated with the other predictor variables; for example, mean annual temperature was excluded because it was correlated with mean minimum temperature, which was the more physiologically relevant variable. Percent urban land cover was inversely correlated with percent vegetation land cover, so we only used percent vegetation based on our hypotheses related to the urban heat island impacts on the thermal environment for rats. We also did not include mean maximum temperature, which was associated with the long-term temperature warming based on well-established latitudinal trends. We did retain two pairwise variables that are correlated because of long-established correlative links. First, human population density is correlated with GDP, which is a well-known association in economics due to urban scaling and Zipf’s Law (scaling based on disparity in city size). For example, Ribeiro *et al.* (*68*) found that across >5000 cities in 96 countries, GDP consistently associates with human population due to that scaling. Second, human population density is correlated with long-term temperature warming, and this pattern is known to be coincident on the basis of urbanization geography and anthropogenic heat emission (*69*) rather than at the subregional scale that most of our temperature data were collected at.

We also calculated the urban and vegetated land cover in each city to look for an association with land cover and rat trends. We obtained these global data as raster images from the European Space Agency (ESA) through their Climate Change Initiative (CCI) and analyzed them in QGIS mapping software. These data were 300-m resolution and included a time series from 1992 to 2020. We added the municipal boundaries for all 16 cities to this QGIS project, added a buffer of 1 km (to conjoin all noncontiguous city parcels), and then used the Zonal Statistics tool to calculate the number of cells of each land cover type. For the ESA-CCI data, we collated the land cover classes that had tree and shrub cover, herbaceous cover, and shrubland (i.e., cover class codes 12, 40, 60-62, 70-72, 80-82, 90, 100, 110, 120-122, 160, 170, and 180) in a “vegetation” category, and the “urban areas” class (code 190) into an “urban” category. We then calculated the proportion of each city that had vegetated and urban land cover. Low percentages of vegetation within cities could be explained by the low spatial resolution of the raster leading to the vegetation being outweighed by urban land covers in the raster’s classification at the 300 m–by–300 m resolution.

The trend in the number of sightings for each city was analyzed using a Mann-Kendall test, which analyzes data for the magnitude of trends in time series data. The *z* statistics were calculated for each city to analyze the magnitude, direction, and significance of the rat trends. The Mann-Kendall test is a nonparametric analysis that does not assume a priori distributions or homoscedasticity and is not sensitive to outliers or nonnormally distributed data (*70*, *71*). We performed a relative importance (or weights) analysis to partition variance among the five environmental variables and quantify their association with the trend *z* statistic. Relative weights analysis is based on the standard regression framework but accounts for multicollinearity among the explanatory variables (*72*). We ran this in the “relaimpo” package within R. Lastly, we performed linear regression between the *z* statistic and each of the five variables to estimate the correlation coefficient for each relationship.
