# Supplementary material for: Increasing rat numbers in cities are linked to climate warming, urbanization, and human population
Source: Sci Adv. 2025 Jan 31;11(5):eads6782. doi: 10.1126/sciadv.ads6782 (PMC11784805; doi:10.1126/sciadv.ads6782)
Supplement: Supplementary file 1 — Table S1 [file sciadv.ads6782_sm.pdf]

Science Advances
AAAS

Supplementary Materials for

Increasing rat numbers in cities are linked to climate warming, urbanization, and human population

Jonathan L. Richardson *et al.*

Corresponding author: Jonathan L. Richardson, jrichardson@richmond.edu

Sci. Adv. **11**, eads6782 (2025)
DOI: 10.1126/sciadv.ads6782

This PDF file includes:

Table S1

Supplementary Materials

Table S1: Supplemental sources for the data from 16 cities used to evaluate trends in rat numbers and associated environmental variables.

City	Data Source	Database or Search Terms	Month/Year Range of Data	Long-Term Temperature Change	Population Size (millions)	Population Density (persons per square km)
Amsterdam	Municipal Health Service (GGD)	Data from complaints with staff visits and confirmed active rats signs	Apr 2009 – Dec 2021	1.72°C (Royal Netherlands Meteorological Institute 2014)	0.908	5214
Boston	Analyze Boston	Rodent Activity; Pick up dead rodent; Rat Bite; Dead rodent	Jul 2011 – Dec 2021	1.733°C (National Oceanic and Atmospheric Administration (via EPA Climate Change Indicators and CONUS Climate Divisions)	0.675	5396
Buffalo	Open Data Buffalo	Rodent_Pest Control; Rodent Control	Jul 2008 – May 2022	1.211°C (National Oceanic and Atmospheric Administration (via EPA Climate Change Indicators and CONUS Climate Divisions)	0.278	2661
Chicago	Chicago Data Portal	Rodent Baiting/Rat Complaint	Jan 2011 – Dec 2022	0.957°C (National Oceanic and Atmospheric Administration (via EPA Climate Change Indicators and CONUS Climate Divisions)	2.746	4656
Cincinnati	Open Data Cincinnati	Rats, problem/infestation; Food operation, rodents; Rats in sewer, rem req; Rats, childrens hosp referral; Rats, in a building; Rats, outside a building; School, rodents in or around; Rodent/insect infest, hotel/mt; Rodent/insect infest, nurse hm	Jan 2012 – Dec 2021	0.742°C (National Oceanic and Atmospheric Administration (via EPA Climate Change Indicators and CONUS Climate Divisions)	0.309	1532
Dallas	Dallas Open Data	Rodents/Vector Control	Oct 2013 – Dec 2019	0.324°C (National Oceanic and Atmospheric Administration (via EPA Climate Change Indicators and CONUS Climate Divisions)	1.304	1482

DC	Open Data DC	Rat Abatement; Rodent Inspection and Treatment	Apr 2009 – Jul 2022	1.381°C (National Oceanic and Atmospheric Administration (via EPA Climate Change Indicators and CONUS Climate Divisions)	0.689	4361
Kansas City	Open Data KC	Health Code Violation: Rats; Rat Treatment	Jan 2007 – Apr 2022	0.586°C (National Oceanic and Atmospheric Administration (via EPA Climate Change Indicators and CONUS Climate Divisions)	0.508	623
Louisville	Louisville Metro Open Data	Rats, Private Property; Rodent/Rat Complaint	Jan 2007 – Jun 2019	0.432°C (National Oceanic and Atmospheric Administration (via EPA Climate Change Indicators and CONUS Climate Divisions)	0.633	752
New York City	NYC Open Data	Rat Sighting	Jan 2010 – Jul 2022	1.989°C (National Oceanic and Atmospheric Administration (via EPA Climate Change Indicators and CONUS Climate Divisions)	8.805	11313
New Orleans	New Orleans Mosquito, Termite, and Rodent Control Board	Rodent Complaint - Rats	Jan 2014 – Feb 2024	0.785°C (National Oceanic and Atmospheric Administration (via EPA Climate Change Indicators and CONUS Climate Divisions)	0.384	875
Oakland	Alameda County Department of Environmental Health	Rat Sighting	Jan 2006 – Aug 2023	1.50°C (National Oceanic and Atmospheric Administration (via EPA Climate Change Indicators and CONUS Climate Divisions)	0.441	3041
St. Louis	St. Louis Open Data	Rats (exterior); Rat Infestation; Rats/Mice Food Ctrl	Jan 2013 – Jul 2022	0.541°C (National Oceanic and Atmospheric Administration (via EPA Climate Change Indicators and CONUS Climate Divisions)	0.301	1886
San Francisco	SF Open Data	Infestation_Rodent_Insect	Jan 2010 – Jul 2022	1.501°C (National Oceanic and Atmospheric Administration (via EPA Climate Change Indicators and CONUS Climate Divisions)	0.873	7194
Tokyo	Tokyo Metropolitan Government Bureau of Public Health and Medical Care	Section 11, brown rats, roof rats, unclear	Apr 2008 – Mar 2021	1.19°C Japanese Meteorological Agency; Synthesis Report on Observations, Projections and Impact Assessments of Climate Change, 2018	13.988	6363
Toronto	Toronto Open Data	Rats, within Property Standards Complaints	Jan 2011 – May 2023	1.81°C Mohsin and Gough 2010	2.8	4427
